# Prevalence of HPV infection in hypopharyngeal and laryngeal squamous cell carcinoma at Thailand's largest tertiary referral center

**DOI:** 10.1186/s13027-017-0167-0

**Published:** 2017-11-02

**Authors:** Warut Pongsapich, Nitathip Eakkasem, Sontana Siritantikorn, Paveena Pithuksurachai, Kshidej Bongsabhikul, Cheerasook Chongkolwatana

**Affiliations:** 1 Department of Otorhinolaryngology, Faculty of Medicine Siriraj Hospital, Mahidol University, Bangkok, 10700 Thailand; 2 Department of Microbiology, Faculty of Medicine Siriraj Hospital, Mahidol University, Bangkok, 10700 Thailand

**Keywords:** Thailand, Prevalence, Human papillomavirus, HPV infection, Laryngeal and hypopharyngeal squamous cell carcinoma

## Abstract

**Background:**

Following the well-established relationship between human papillomavirus (HPV) and cervical carcinoma, the carcinogenicity of this virus has also been confirmed in subsets of head and neck carcinoma (HNCA), but mainly in the oropharynx. Other subsites of HNCA with less known association to HPV have never been studied in Thailand. Accordingly, the aim of this study was to investigate the prevalence of HPV DNA in hypopharyngeal and laryngeal squamous cell carcinoma in Thai population.

**Methods:**

This cross-sectional study included hypopharyngeal and laryngeal squamous cell carcinoma patients diagnosed and treated at the Department of Otorhinolaryngology, Siriraj Hospital during the September 2011–December 2013 study period. Presence of HPV genome was confirmed by polymerase chain reaction from pathologically-confirmed fresh specimens. Demographic data and risk factors of HPV infection were evaluated.

**Results:**

Eighty patients were included, and 95% of those were male. Only one patient was noted with positive HPV-62 serotype. Most patients consumed tobacco and/or alcohol. Five patients had no risk factors for cancer development. Risk of HPV infection was evaluated by self-reporting questionnaire. The mean age of sexual debut was 20.17 years. Forty-eight patients had multiple sexual partners. Sixteen and seven patients had history of sexually transmitted disease infection and habitual oral sex contact, respectively.

**Conclusion:**

There was no oncogenic HPV DNA detected within pathologic specimens of laryngeal and hypopharyngeal cancers in this study. Compared to rates reported from developed countries, the prevalence of HPV-related HNCA in Thailand is very low.

## Background

The incidence of head and neck cancer (HNCA) is currently declining. In 2012, the World Health Organization (WHO) estimated 686,000 new cases of HNCA [1]. comprising 300,000 cases of oral cavity cancer, 142,000 cases of pharyngeal carcinoma, and 157,000 cases of laryngeal carcinoma. HNCA-related deaths were estimated to be 375,000 cases per year. Moreover, the burden of cancer has gradually shifted to less developed countries which now account for 65% of cancer deaths worldwide [1].

Certain lifestyle behaviors, such as smoking, poor diet, and physical inactivity, are known major risk factors for carcinogenesis. Other possible risk factors for the upper aerodigestive malignancies include chronic mucosal irritation and gastroesophageal reflux disease. Infection has been acknowledged as an etiological factor in certain types of cancer, including stomach, liver, and cervical cancer. In HNCA, human papillomavirus (HPV) infection has been proven to be a major cause of cancer development only at the base of tongue and in the tonsillar regions. Nonetheless, due to the heterogeneity of HNCA, the relationship between HPV infection and other subsites of the head and neck has not been conclusively established.

The global impact of HPV-related malignancies has been analyzed in accordance with criteria set forth by the International Agency for Research on Cancer. A strong association has been identified between HPV and HNCA in high Human Development Index (HDI) regions and countries, including Europe, North America, Australia, New Zealand, and Japan [2]. The incidence of HNCA is disproportionate among ethnic groups, with the highest prevalence in non-Hispanic white men [3]. HPV related cancer is also more prevalent in people with high socioeconomic status, high education and young patients [4]. Despite typical cervical node involvement, the prognosis in these demographic subsets is excellent.

Even though HPV infection can develop in almost all parts of the upper aerodigestive tract, the classical manifestation is often located at the larynx. Laryngeal papillomatosis is a benign pathologic lesion that has been recognized for almost a century. The larynx is usually infected by low oncogenicity HPV subtypes that have a malignant transformation rate of less than 2% [5]. A systematic review by Kreimer*,* et al. found 24% prevalence of HPV in more than 1400 laryngeal or hypopharyngeal cancer patients using the polymerase chain reaction (PCR)-based method [6].

Subsites of HNCA with less known association to HPV than the oropharynx have never been studied in Thailand. Accordingly, the aim of this study was to investigate the prevalence of HPV DNA in hypopharyngeal and laryngeal squamous cell carcinoma at Thailand’s largest national tertiary referral center.

## Methods

This cross-sectional study included hypopharyngeal and laryngeal squamous cell carcinoma patients diagnosed and treated at the Department of Otorhinolaryngology, Siriraj Hospital, Thailand’s largest national tertiary referral center, during the September 2011 to December 2013 study period. The protocol for this study was approved by the Siriraj Institutional Review Board (SIRB), Faculty of Medicine Siriraj Hospital, Mahidol University, Bangkok, Thailand.

Patients aged >20 years that were diagnosed with laryngeal or hypopharyngeal squamous cell carcinoma without prior treatment during the study period were included. Patients were divided into one of two study groups according to cancer type. Laryngeal cancer was subclassified into three subsites, including supraglottic, glottic, and subglottic regions. Hypopharyngeal cancer was subdivided into pyriform sinus, posterior pharyngeal wall, and postcricoid regions. Both cancers were staged according to the seventh edition of American Joint Committee on Cancer (AJCC) staging criteria [7]. The sample size of 40 patients per cancer group was calculated based on the 24% prevalence of HPV-positive laryngeal and hypopharyngeal cancer reported by Kreimer, et al. [6].

The case record form used for this study was divided into two parts, as follows: Part 1) Demographic data and clinical information; and, Part 2) Data from the concealed self-reporting Thai-language questionnaire about behavioral risk factors for HPV infection and cancer development, including number of lifetime sexual partners, age of sexual debut, history of habitual oral sex practice, and history of sexually transmitted diseases. Patients were notified of their right to decline answering the sexual behavior questions listed in part 2.

A 1.5–2 mm histologically-confirmed fresh specimen of hypopharyngeal and laryngeal squamous cell carcinoma from each included patient was prepared by pathologist. DNA was extracted, and PCR of linear array hybridization for specific HPV test was performed in all 80 specimens. Biotinylated DNA was used as a primer for genotypic testing. The protocol used for HPV identification was previously described by Steinau et al., with 96% sensitivity and 99% specificity [8]. Oncogenic types of HPV included type 16, 18, 31, 33, 35, 39, 45, 51, 52, 56, 58, 59, 68, and 73 [9, 10].

## Results

Eighty patients were included, and 95% of those were male. There were 40 patients in the hypopharyngeal group and 40 patients in the laryngeal group. The mean age of patients was 61.5 years (range: 34–89). More than 80% of patients presented with an advanced stage, and almost all of them were smokers (Table 1). Pyriform sinus was the most common subsite of hypopharyngeal cancer, while laryngeal cancer predominantly originated in the glottic region.

**Table 1:** Patient characteristics of laryngeal and hypopharyngeal cancer

	Total cases (%)
Gender	
Male %	76 (95)
Female %	4 (5)
Risk	
Alcohol drinking	58 (72.6)
Tobacco smoking	73 (91.3)
Stage	
Early (stage1–2)	15 (18.75)
Advance (stage 3–4)	65 (81.25)

More than 70 patients (87.5%) completed the questionnaire that elicited data about risk behaviors for HPV infection (Table 2). There was only one positive HPV DNA finding from all pathologic specimens. That positive specimen was taken from a 72-year-old male patient with T2N0M0 pyriform carcinoma. He had no risk factors for HPV infection, but he reported habitual smoking and drinking behavior. HPV-62 serotype was identified in subtype analysis, yet no oncogenic virus was found within the tumor specimen.

**Table 2:** Risk of HPV infection

Risk of HPV infection (percent)	Case (%)
Age of sexual debut	
< 20 years	53/71 (74.6)
> 20 years	18/71 (25.4)
Sexual partner	
Multiple partners	48/73 (65.8)
Single partner	25/73 (34.2)
Habitual oral sex activity	
Yes	7/70 (10)
No	63/70 (90)
History of sexually transmitted diseases	
Yes	16/75 (21.3)
No	59/75 (78.7)

## Discussion

HPV-positive malignancies represented 5–20% of all HNCA [11]. Data from the US and Europe showed 40–80% and 20–90% of those HPV infections arising from the oropharynx, respectively [11–13]. This prevalence was relatively high, compared to our previous study [14].

In our previous study, only 6% of HPV-related cancer was found in the oropharynx. In this present study, no oncogenic HPV infection was identified in pathologic specimens of laryngeal and hypopharyngeal cancer among Thai study population. This discrepancy in HPV prevalence might be due to a very high rate of tobacco-derived carcinogenesis in our population, which is similar to the result reported from a study in Northern Spain [15]. The patients described in low prevalence reports appear to share similar characteristic. Specifically, most were non-white men in their seventh decade of life, with low socioeconomic status and advanced tumors [16, 17].

HPV is transmitted sexually through micro-abrasions of skin or mucosa. As a subclinical infection, HPV is the most common sexually transmitted infection worldwide, with an estimated lifetime risk of cervical HPV infection in women of up to 80% [18]. Viral genome is mainly confined to the basal cell layer of the junction between squamous and columnar epitheliums. Presence of HPV infection in head and neck region may be related to high-risk sexual behaviors, including early age of sexual activity, number of lifetime sexual partners, habitual oral sex practice, and history of other sexually transmitted diseases [19, 20]. Moreover, the transmission rate might increase with an inflammation, such as tonsillitis or pharyngitis due to micro-disruption of mucosal membrane.

The low prevalence of HPV infection in our study population may reflect a conservative culture of Asian countries during the past 50 years. With regard to the sexual behavior questionnaire, it is possible that some of the reported data was inaccurate. For example, the age at first sexual activity and the number of life time partners for some study respondents may have represented only a ‘best guess’ estimate. It should also be noted that detection of HPV genome does not conclusively indicate past high-risk sexual activity. As such, the collection of previous sexual behavior data may not have been beneficially informative in this study.

The first reason why HPV may be undetectable in non-oropharyngeal squamous cell cancer is that different mucosal linings have different characteristics. While the palatine and lingual tonsils of the base of the tongue, which are parts of Waldeyer’s ring, are mostly composed of lymphoepithelial tissues, the hypopharynx is lined with columnar epithelium. The other reason is that most of the human papillomavirus could be cleared by intact immunity without causing any clinical manifestations. Only in a small number of people, viral genome can integrate into host tissue and remain in a latent form, and then can progress later into an invasive lesion.

The overall rate of high risk HPV infection detected in oral rinsed exfoliated cell among sexually active adults in the US was reported to be 3.7% [21]. There is currently no consensus regarding which technique for diagnosing HPV should be used in HNCA. The sensitivity and specificity of each method are affected by the concentration of the HPV infection and the quality of diagnostic specimen. In the current study, real-time polymerase chain reaction (PCR) was used to detect very small copies of viral DNA. Moreover, primers were designed to target highly conserved sequences shared by multiple HPV types, which facilitated the identification of multiple HPV subtypes. Despite the high sensitivity of this method, the positive result did not identify the transcriptional activity of viral DNA [22].The HPV type 62 detected in the hypopharyngeal specimen in this study is categorized as a low-risk subtype with a non-carcinogenic HPV classification. It is only a passenger virus of transcriptional silence and plays no role in the process of tumorigenesis.

In situ hybridization(ISH) is a preferred method for determining the integration of viral genome into a host cell. To cover a broader range of viral subtypes, HPV probe cocktails have recently been invented. This method has higher specificity, lower sensitivity, is more time consuming, and is only available in selected centers [23]. Transcriptional activation of viral oncoproteins E6 and E7 is generally regarded as the gold standard method for confirmation of HPV status. The detection of E6/E7 mRNA is required for the sophisticated technique of RNA extraction followed by PCR amplification. Given that this method is technically challenging and has poor reproducibility, it is generally restricted to the research laboratory [23, 24].

As a result of its high positive predictive value, overexpression of p16 protein is proposed as a surrogate marker of HPV activation in oraopharyngeal cancer. Strong staining of p16 in classic HPV-related histomorphology is more practical and cost effective than PCR and ISH [25]. In contrast, without HPV prevalence, the accuracy of p16 immunohistochemistry is low. In cases where the presence of HPV needs to be conclusive determined, more accurate HPV testing methods are indicated.

Laryngeal papillomatosis is the most common benign neoplasm of the larynx. The HPV genome is integrated at the squamocolumnar junction of the true vocal cord. Most infections are caused by noncarcinogenic viral strains type 6 and 11. No strong evidence has yet been reported that establishes association between HPV infection and laryngeal cancer. Papilloma viral tropism reflects a distinct host-viral interplay among different virus subtypes. A phylogenetic study reported that each variant has its own distinctive potential to induce lesions among different types of mucosal epithelium [26]. Consistent with that reported finding, certain HPV16 subtypes are specifically associated only with vulvar cancer, while cervical cancer is associated with other types of high-risk HPV, such as types 18, 31, and 33 [27].

In our study, the HPV from the patient who was found to be positive for HPV type 62 serotype belongs to the *Alphapapillomavirus* genus of viruses. HPV-62 is a low-risk HPV that was reported as coinfection in 5.1% of the HPV-positive cervical samples [28]. In India and Egypt, HPV type 62 is the most prevalent type among low-risk types of HPV [29, 30]. Similarly, in unvaccinated Thai women, HPV-62 is the second most frequent HPV detected in the cervical screening program [31]. Regardless of its prevalence, HPV type 62 has never been reported in HNCA worldwide.

The patients enrolled in this study were from a single center, Thailand’s largest tertiary referral hospital, which means that we are often referred patients with complicated and intransigent conditions. As such, it is possible that our findings may not be generalizable to patients with the same condition in other settings.

## Conclusion

The epidemiological trends of head and neck cancer can be classified into two groups, HPV-related and HPV-unrelated HNCA. In recent decades, with the increasing prevalence of HPV-related HNCA, the overall prevalence of HPV-unrelated HNCA has gradually been decreasing due to the reduction of tobacco and alcohol consumption. Hence, HPV status is proved to be an effective prognostic predictor that can potentially affect the outcome of treatments. Particularly, identification of HPV is mandatory in the high prevalence of anatomical subsite.

In this study, only 1 of 80 cases (0.0125%) had positive HPV DNA finding. HPV-62 serotype (non-oncogenic) was identified. There was no oncogenic HPV DNA detected within pathologic specimens of laryngeal and hypopharyngeal cancers.

Since any subsites other than oropharynx are considered as HPV-unrelated, the detection is thus not routinely recommended in our institute. Moreover, the risks of sexually transmitted diseases in the past do not determine the possible risk of cancer development, especially in countries with less strong association of HPV driven carcinogenesis.

## Abbreviations

HNCA: Head and neck cancer
HPV: Human papilloma virus
